# Design and Performance Evaluation of an Algorithm Based on Source Term Estimation for Odor Source Localization †

**DOI:** 10.3390/s19030656

**Published:** 2019-02-05

**Authors:** Faezeh Rahbar, Ali Marjovi, Alcherio Martinoli

**Affiliations:** Distributed Intelligent Systems and Algorithms Laboratory, School of Architecture, Civil and Environmental Engineering, École Polytechnique Fédérale de Lausanne (EPFL), 1015 Lausanne, Switzerland; ali.marjovi@epfl.ch (A.M.); alcherio.martinoli@epfl.ch (A.M.)

**Keywords:** odor source localization, source term estimation, mobile robotics

## Abstract

Finding sources of airborne chemicals with mobile sensing systems finds applications across safety, security, environmental monitoring, and medical domains. In this paper, we present an algorithm based on Source Term Estimation for odor source localization that is coupled with a navigation method based on partially observable Markov decision processes. We propose a novel strategy to balance exploration and exploitation in navigation. Moreover, we study two variants of the algorithm, one exploiting a global and the other one a local framework. The method was evaluated through high-fidelity simulations and in a wind tunnel emulating a quasi-laminar air flow in a controlled environment, in particular by systematically investigating the impact of multiple algorithmic and environmental parameters (wind speed and source release rate) on the overall performance. The outcome of the experiments showed that the algorithm is robust to different environmental conditions in the global framework, but, in the local framework, it is only successful in relatively high wind speeds. In the local framework, on the other hand, the algorithm is less demanding in terms of energy consumption as it does not require any absolute positioning information from the environment and the robot travels less distance compared to the global framework.

## 1. Introduction

In many situations, finding the source of a gaseous chemical released in the air becomes vital. For instance, robotic olfaction systems might be deployed in search and rescue, or safety and security operations to find explosives or drugs in airports or industrial facilities, or to search for survivors in the case of natural hazards. Despite the growing community of robotics and environmental engineers studying this crucial topic for more than two decades, odor source localization using mobile or static sensor nodes remains a challenging operation in realistic environments. The main difficulty arises from the dispersion phenomenon [1] which is a combination of molecular diffusion that drives odor patches away from the source, as well as the advection due to the airflow that carries the molecules in its direction [2]. As a result, the odor plume is shaped not only by the characteristics of the source, but also by the airflow which, in turn, is influenced by the environment. Further, air flow characteristics such as turbulence, meandering motions, and fluctuations impact the plume structure as well and make it more difficult to traverse.

To simplify the challenging problem of Odor Source Localization (OSL), scientists usually divide it into three sub-tasks [3]: (i) odor plume acquisition, which refers to a search method that aims to find the plume in the environment; (ii) odor plume tracking, which is the phase where the robot leverages the samples obtained from the plume to localize the source; and (iii) odor source declaration, the final task, during which the robot validates and declares the location of the source.

The first and the last sub-tasks usually involve other sensing modalities (such as vision) and depend on the application or the scenario in which the strategy is taking place. Therefore, most studies in the literature focus on the plume tracking sub-task, which is the core and most challenging part of the mission. Various strategies have already been implemented for this sub-task, which can be classified into four often overlapping categories [4]: gradient-based, bio-inspired, formation-based, and probabilistic algorithms.

Gradient-based algorithms assume the plume to be a smooth concentration gradient, which could be the case in an environment without an airflow. For this class of algorithms, the robot needs to sample at different points in the environment while moving slowly to measure the long-term average of odor concentration at each sampling point [5]. Therefore, these algorithms, while being the most intuitive and computationally light ones, need relatively long time windows to find the source.

Bio-inspired algorithms define methods similar to existing behaviors in nature, such as search strategies of moths, dogs, bacteria, etc. [6]. Casting, Surge-Cast and Surge-Spiral [7,8,9] are among the most successful ones in this class that are inspired from moths behavior. The advantage of these algorithms is their independence from any a priori information about the environment, its atmospheric conditions, or the historic observations, which makes them robust, though not necessarily efficient, for unknown and dynamic areas. Nevertheless, due to low performance of current sensing and locomotion technologies compared to their biological counterparts, these algorithms are still far from being reliable in realistic environments [10].

In a relatively new approach, formation-based algorithms are natively designed for multi-robot systems [11]. Sampling multiple points at the same time is an advantage of this type of method, but since the coordination between agents is necessary, the entire method relies on inter-robot communication and relative positioning.

Probabilistic algorithms model a belief on the source location in the form of a probability distribution derived from the observations made by the agents in the environment [4]. After each observation, the belief is updated using Bayesian estimation. This process continues until the probability distribution reduces to a Dirac function. Infotaxis [12], particle-based algorithms [13], and Source Term Estimation (STE) [14] are the main examples of this category.

While probabilistic algorithms can be computationally expensive, they have many advantages. Firstly, unlike the previously mentioned methods that provide only the source position, they usually generate a richer set of information about the environment (e.g., [15]) or the source characteristics [14]. Secondly, since the framework is probabilistic, the provided information is associated with an amount of uncertainty, which shows how trustworthy the data are. Moreover, unlike the other algorithmic classes, the sensing agent does not necessarily have to physically approach the source to localize it, hence the process is faster and remains applicable in situations where the source is inaccessible. Lastly, compared to the other categories, probabilistic algorithms are more flexible in terms of the type of underlying hardware (e.g., static, mobile, and single- or multi-agent).

Most of the probabilistic algorithms rely on a plume model to update their belief on the source position. Since these models need to be customized for each specific environment, they cannot be used in an unknown environment. STE methods, on the other hand, while relying on a mathematical formulation for the model based on the physics of transport phenomena, do not use any a priori information on the parameters of the model, i.e., the characteristics of the plume.

Considering the above-mentioned advantages, we chose to focus on an STE algorithm in this paper. The goal of STE algorithms is to learn the parameters of the source, such as the release rate, the release start and stop time, the dispersion in the space, and, most importantly, the position of the source. The choice of the parameters depends directly on the chosen plume model. The method aims to learn the parameters of the model while the sensory system gathers data in the environment. Since the concept is very broad, the algorithm is not exclusively used for gas sources; depending on the model, it can be applied to any type of source (e.g., radiation [16]). It is also not limited to mobile sensor nodes, as it can be used with a static sensor network (e.g., [17]).

However, when used on mobile robots, STE algorithms can be coupled with a navigation strategy which makes the data collection more time-efficient. Different navigation methods have already been used with STE in the literature. Partially Observable Markov Decision Processes (POMDP) [16] and mutual information maximization [18] are among the popular methods in this area. However, most of the works involving mobile robots have only been evaluated in simplified simulations (e.g., [18,19,20]).

Hence, the contributions of this work are the following:

- An enhanced navigation strategy is presented for an STE algorithm and was evaluated in a high fidelity robotics simulation software as well as in a controlled physical set-up.
- To make the evaluation suitable for unknown environments, the algorithm is developed for two different frameworks, one with global knowledge about the environment and the other with local knowledge.
- A systematic performance evaluation was carried out to verify the robustness of the method to various environmental conditions.
- The influence of a few key algorithmic parameters of the method on the performance was also studied.

The remainder of this paper is stuctured as follows. We present the details of the algorithm in the next section, and then we explain the evaluation process and present the obtained results. Finally, we discuss the outcome of the experiments before we conclude and present the outlook for this work.

## 2. Methods

As discussed in the Introduction, this work is built on the STE algorithm. STE is a probabilistic framework in which different strategies can be used. The general idea is to rely on a plume concentration model whose parameters have to be estimated throughout the experiment. The nature of the parameters depends on the model of choice, but, in any case, they include the source position, which is the goal of the OSL problems.

STE methods are usually flexible enough to be performed on a large variety of sensing assets, ranging from static sensor networks to heterogeneous mobile robots. In the case of a mobile system, the estimation algorithm is augmented with a navigation strategy to increase the amount of acquired information at each step.

Since in most of the applications of OSL the optimal method is the one with the lowest time of estimation, we believe that using mobility could significantly improve the performance. Moreover, assuming slowly changing plume conditions, in absence of mobility, a large number of static sensors would be required to capture spatially rich enough information, while much fewer mobile sensing assets would be sufficient to gather the same amount of information. Therefore, in this work, we use a single mobile robot equipped with a chemical sensor to sample the environment.

**Figure 1:**
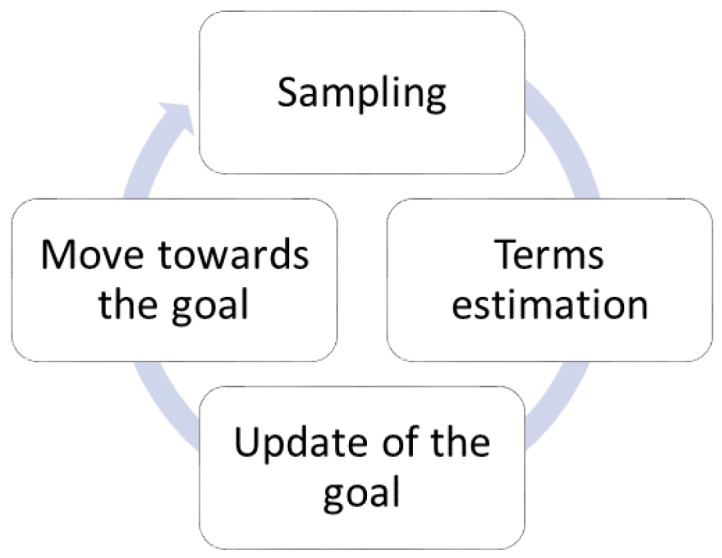
The global structure of STE algorithm using a mobile robot.

As a result, our method consists of two main parts: estimation and navigation. In the estimation part, the algorithm estimates the parameters using the data that the robot has acquired. In the navigation part, the goal is to send the robot to the best neighboring point to obtain as much information as possible. This cycle, represented in Figure 1, continues until the uncertainty on the parameter estimation becomes negligible.

### 2.1. Local vs. Global Frameworks

This concept is applied in two different frameworks that we call global and local. In the global framework, it is supposed that, firstly, the map of the environment is available for the robot, and, secondly, the robot can localize itself using a global positioning system. In the local framework, on the other hand, the robot has no information about the map of the environment and does not have access to global positioning information. Therefore, it creates a fixed-size local map (e.g., $2\times 2\;m^{2}$ in this paper) aligned with the wind direction around itself and performs the estimation and navigation on that local map using odometry. Once enough information is acquired on the local map, it is then moved to another place.

Although the global framework seems more practical and easy to implement, we believe that the local framework is more realistic for unknown environments without a predefined map and availability of a global positioning system.

Even though the two frameworks seem very different in terms of localization method, we applied the same concept on both of them and there are only a few minor differences in the implementation. In the remainder of this section, we discuss the details of the method in both frameworks.

The method as well as additional results concerning the global framework are reported in a further conference paper [21].

### 2.2. Plume Model

Probabilistic algorithms usually rely on a plume model for updating their belief about the source position. In this work, we chose to use the pseudo-Gaussian concentration plume model [22], presented in Equation (1), which describes the time-averaged concentration model for a continuous point source in a laminar flow, where *Q* is the source release rate and $\bar{u}$ the average wind speed.

(1) $$C\left(Q,x_{s},y_{s},z_{s},\sigma_{y},\sigma_{z}\right)=\frac{Q}{2\pi \bar{u}\sigma_{y}\left(x-x_{s}\right)\sigma_{z}\left(x-x_{s}\right)}e^{-\frac{{\left(y-y_{s}\right)}^{2}}{2\sigma_{y}^{2}\left(x-x_{s}\right)}-\frac{{\left(z-z_{s}\right)}^{2}}{2\sigma_{z}^{2}\left(x-x_{s}\right)}}\;\forall x\geq x_{s}$$

In this equation, the *X*-axis is assumed to be aligned with the direction of the airflow and it is defined for all the x downwind of the source (i.e., $\forall x\geq x_{s}$). The concentration is simply 0 for all points upwind of the source (i.e., $\forall x<x_{s}$). The source is positioned at $\left(x_{s},y_{s},z_{s}\right)$ and $\sigma_{y}$ and $\sigma_{z}$ are the standard deviations of odor dispersion in the *Y*- and *Z*-axis, respectively; they are simplified to be linear functions of upwind distance from the source $\left(x-x_{s}\right)$.

Moreover, since we are leveraging an STE method, the goal of the algorithm is to estimate the parameters of the model. Therefore, the set of parameters to be estimated for this model is $m=\{Q,x_{s},y_{s},z_{s},\sigma_{y},\sigma_{z}\}$. As the standard deviations $\sigma_{y}$ and $\sigma_{z}$ are both of form $\sigma \left(x-x_{s}\right)=a\left(x-x_{s}\right)+b$, they each have two parameters to be estimated. Therefore, to simplify further, the plume is assumed isotropic in *Y* and *Z* directions, hence $\sigma_{y}=\sigma_{z}$. Thus, after the simplifications, six parameters remain to be estimated, making the problem six-dimensional.

### 2.3. Parameter Estimation

The estimation is performed probabilistically using the Bayesian formulation presented in Equation (2), where *m* represents the set of model’s parameters and *D* the obtained data through sampling. The posterior P(m|D) represents the probability distribution on the parameters values.

(2) $$P\left(m|D\right)=\frac{P(m)P(D|m)}{P(D)}$$

The evidence P(D) being a normalization factor, it can be neglected. Hence,

(3) P(m|D)∝P(m)P(D|m)

We consider the prior P(m) a uniform distribution in between the limits of each parameter. Therefore, the posterior P(m|D) will be proportional to the likelihood P(D|m) in the parameters limit, and equal to 0 outside.

The likelihood P(D|m) defines the probability of obtaining a set of data, given a set of parameters. In other words, it returns the likelihood of a set of parameters given the data that the robot collected up to the present time. It is defined in [17] as follows:

(4) $$P\left(D|m\right)\propto exp\left(-\frac{1}{2}\sum_{i}\left(\frac{{\left(D_{i}-C_{i}\left(m\right)\right)}^{2})}{\sigma_{M}^{2}+\sigma_{D}^{2}}\right)\right)$$

where $\sigma_{M}$ and $\sigma_{D}$ represent the standard deviations of model and measurement error, respectively. Both errors are assumed to be normally distributed, with mean on 0.

The sum in Equation (4) is applied on all samples the robot gathered during the experiment. $C_{i}\left(m\right)$ is the concentration determined by the plume model for a set of source parameters *m* for sample *i*. It is defined using the pseudo-Gaussian concentration plume model presented in Section 2.2. $D_{i}$ is the actual measured data for the very same sample *i*. Since we use a time-averaged plume model, the sample needs to be represented by the mean of the sensed concentrations over a fixed time window. Therefore, to gather one sample, the robot stops at each target point for 5 s and then calculates the average of the sensed values gathered at 10 Hz (50 concentration values in total).

Since there are six parameters to estimate, the posterior probability density function has to be a six-dimensional matrix, which is very time consuming to be entirely calculated. The solution to this problem would be to use an approximation algorithm such as the Markov Chain Monte Carlo (MCMC) [23] that allows evaluating the posterior probability function through efficient sampling.

In this work, we use the Metropolis–Hasting method [24]. The important factors in this method are the number of iterations and the proposal distribution, which is chosen as a 6-D Gaussian function, since the posterior probability function is also 6-D. Here, we only present the concept from a general perspective.

At each iteration, a candidate point *p* in the proposal distribution is chosen, which is equivalent of a set of parameters $p=\{Q_{p},x_{p},x_{p},z_{p},a_{p},b_{p}\}$. Then, the likelihood of this point P(p|D) is calculated and compared to the one of the previous point. The higher is the likelihood of the new point compared to the previous one, the higher is the chance of accepting it. In the case it is accepted, its likelihood is saved on the posterior probability distribution and replaces the previous point as the mean of the proposal distribution.

In both frameworks, the estimation part is performed identically. The only difference would be the size and granularity of the posterior probability distribution on which the estimation takes place. More precisely, in the global framework, the range of the parameters related to the source position is based on the whole environmental map, while, in the local framework, it is defined by the limits of the local map. The range of the other parameters are equally chosen in both frameworks.

### 2.4. Navigation

The goal of the navigation method is to feed the estimation part with worthwhile information. Therefore, it needs to predict which point is rich in terms of information to send the robot to. For this purpose, we propose to use POMDP [25] as a predictive navigation method to guide the robot in a profitable fashion.

POMDP requires three components for its operation: a posterior distribution, a set of possible actions, and a reward function. The posterior distribution is already given by the estimation part. As for the set of actions, to simplify the robot’s motion, we limit the movement on one axis at a time. Since the movement is possible in both positive and negative directions on all three axes, the set of possible actions becomes six-fold in a 3-D environment. The most essential component of this navigation method is the reward function, as it determines the behavior of the robot. In this work, it is chosen to be the relative entropy (also known as Kullback–Leibler divergence) [26] which represents the gain of information from one probability density function *P* to another *Q*, defined in Equation (5). In our case, *P* would be the probability density function obtained through the estimation part, and *Q* the predicted probability density function that is calculated separately for each of the target points.

(5) $$D_{KL}\left(P||Q\right)=\sum_{j}P\left(j\right)log\frac{P(j)}{Q(j)}$$

Thus, at each step, once the six possible target points are determined, the robot will first predict the concentration that would be measured in each of them, using the current estimation of the model parameters. Then, it calculates the potential update of the posterior probability function using the Bayesian formulation, which would result in the predicted posterior probability function *Q*. Finally, it evaluates the gain of information at each point using Equation (5).

The chosen target point leads the robot to the direction with the best gain of information. However, in the case where the robot does not have any information about the plume, i.e., no concentration is sensed, no direction would give more information than others. Hence, the robot tends to serially sample in all directions, and, as a result, it stays in the same region for a long time. To make the search more global, we need a component in the navigation strategy that promotes more exploration at the beginning, as well as leads the robot gradually towards the source when the estimation becomes more accurate. This indicating index can be seen in the evolution of the maximum a posteriori value of the source position.

Indeed, while the value of entropy is around maximum (i.e., very little information is available), the maximum a posteriori value, remains very unstable, but it converges towards the ground truth, similarly to the expected value, when the estimation becomes more accurate.

Therefore, we suggest to define the movement vector as a weighted sum of two 3-D vectors: the one leading towards the target point that provides more information, given by Kullback–Leibler divergence calculation, ${\vec{V}}_{KLD}$ and the vector that leads to the maximum a posteriori value of the source position ${\vec{V}}_{source}$.

(6) $$\vec{V}=\alpha {\vec{V}}_{KLD}+\left(1-\alpha \right){\vec{V}}_{source}$$

Intuitively, one would suggest that the algorithms should lead the robot towards the expected source position, as opposed to the maximum a posteriori. However, the expected source position never promotes exploration and might entrap the robot in an equilibrium. The coefficient α in Equation (6) determines how global or local the search would be. Its optimal value is studied in Section 3.

#### Specifications of the Navigation Method in the Local Framework

The above-mentioned navigation method is valid for both local and global framework. However, in the case of the local framework, we need to introduce additional algorithmic features in order to cope with the lack of global localization and environment map. A summary of all of them can be found in Algorithm 1.

As mentioned above, since the robot does not have the map of the environment, it has to generate a local one as a base for its estimation and navigation. The same procedure as the global framework is then applied to the local map with the exception that it is done in a smaller scale and in different steps. More concretely, the algorithm is run on one local map, and then the map is moved to another place where the robot restarts from the beginning. The cycle continues until the source is localized inside the map and thus the entropy of the posterior probability distribution drops below a fixed threshold.

Unlike in the global framework, where the shape of the environment is known for the robot, in the local framework, while navigating towards a target point to sample, the robot might encounter an obstacle, for instance a wall. In this work, for the sake of simplicity, when the robot encounters an obstacle on its way, it simply stops after having carried out an obstacle avoidance maneuver (in our case, we used a simple Braitenberg algorithm [27]), and then sets the measurement of the target point to 0. In this way, that point and its neighboring points are not considered as sampling point candidates. Then, the cycle continues normally.

Another process that is special to the local framework is the way the robot decides to move from one local map to the successive one. Conceptually, this should happen when there is enough information for the robot to move on. However, since the error on odometry accumulates with every step, we wish that the robot resets all position based information (i.e., self-localization and samples positions) in the new local map to reduce the influence of odometry error on the performance. Therefore, moving to a new map means erasing all the previously acquired information. Hence spending too much time in one map would be a waste of time. Spending too little time, on the other hand, although it reduces the number of iterations, could be misleading. As a result, the maximum number of samples in the local map is one important factor that we will study in this paper.

**Figure 2:**
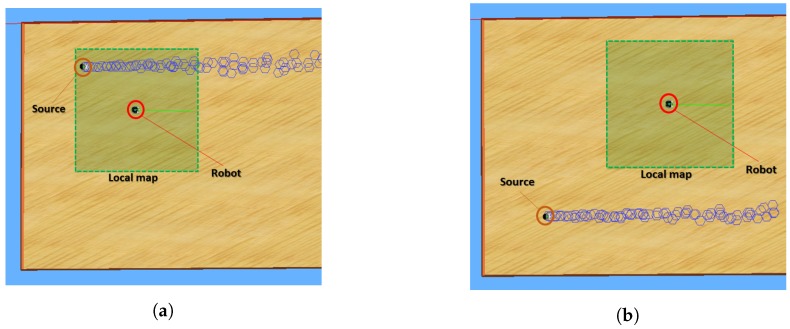
Position of the local map with respect to the source and the plume: (**a**) source inside the local map of the robot and (**b**) local map outside the plume.

Moreover, sampling the same number of points in all the local maps does not appear to be optimal. Depending on where the local map is located with respect to the source location and the plume in general, the robot might need to sample more, or fewer points. The case that illustrates this situation is when the source is located inside the local map. In this situation, we would want the robot to continue sampling in the same map until the end condition is satisfied, rather than move to another map (which will be most likely again around the source) and start over. The opposite case is when the local map is completely outside the plume and the robot would sense no concentration at any point of the map. In this situation, we would like the robot to move on to the next map as soon as possible, where it might have a better chance of obtaining relevant information. Figure 2 illustrates the two mentioned examples.

Based on the arguments above, we adapted the number of samples in each map to the amount of information that we obtain through sampling. More specifically, we define an initial sample budget $B_{0}$ that represents the number of samples that the robot would make if no information was available. The budget is then reduced every time one sample is made. However, this reduction is not the same at every iteration and depends on how valuable the newly obtained sample was. If the sample does not bring any information, i.e., the entropy stays the same as the previous iterations or even increases, the budget is reduced by 1. If the entropy drops, on the other hand, the budget would be reduced by less, depending on the amount of obtained information. Equation (7) shows the mathematical definition of the budget reduction. Note that $H_{previous}$ can never be equal to 0 because, as explain in Section 2.5, as soon as the entropy drops below a certain positive threshold, the algorithm stops. The optimal value of the initial sample budget $B_{0}$ is studied further in this work.

(7) $$B_{t}=\left\{\begin{array}{cc} B_{0} & \;\mathrm{if}\;t=0\\ B_{t-1}-1 & \;\mathrm{if}\;\frac{H_{current}}{H_{previous}}\geq 1\\ B_{t-1}-\frac{H_{current}}{H_{previous}} & \;\mathrm{otherwise} \end{array}\right.$$

**Figure 3:**
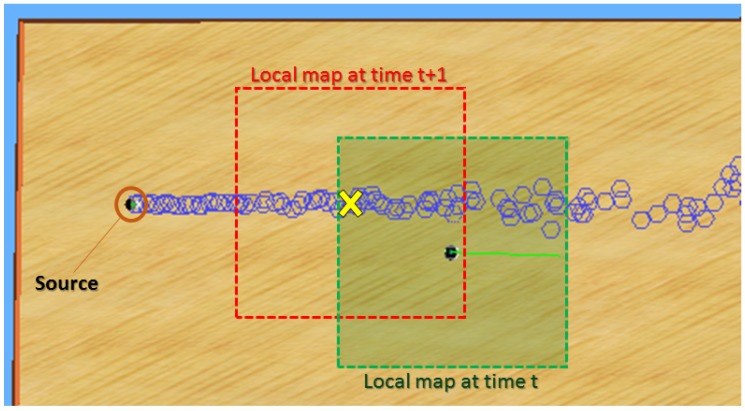
At time *t*, the local mp is located at the position of the green square. The yellow cross shows the maximum a posteriori value of the posterior probability density function, which is the point that is going to become the center of the local map at time t+1. When the robot decides to move the map, it travels to the yellow cross point, and it restarts the procedure in the local map indicated by the red square.

Once we know when to move to a new map, we need to know the best point to move to. For the reasons mentioned in Section 2.4, we believe that maximum a posteriori value of the final posterior probability density function would be the best option. Indeed, we need to explore the environment as much as possible when we receive very little information and we want the robot to get closer to the source step by step when it has enough information. Using relative entropy here would not be useful since, when we move to the new map, the previous points would already be forgotten. Therefore, pure exploitation here seems to be preferable. Thus, when the robot decides to move to a new map, it goes to the point indicated by the maximum a posteriori value of the posterior probability density function, which becomes the center of the new map. From that point, all the procedure starts over. Figure 3 shows a graphical representation of the move from one local map to another.

In the same line of exploitation, when no concentration is sensed in one map, there is still one source of information that can be exploited, which is the wind direction. Therefore, to increase the exploitation, our method moves the map crosswind when the sum of all sensed concentrations is equal to 0. Whether to move left or right along the crosswind direction is chosen randomly in a uniform way, and can be changed at each iteration.

**Algorithm 1:** Local framework: when and how to change the position of the local map.

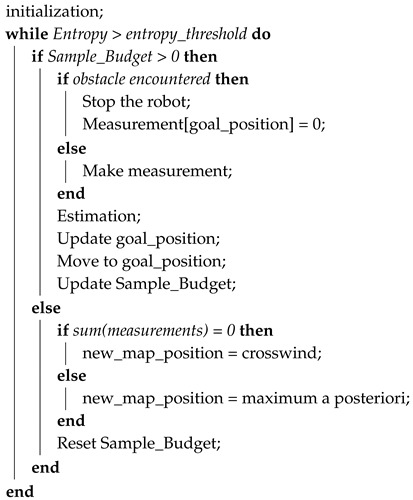



### 2.5. End of Algorithm

As mentioned above, the algorithm stops when the uncertainty on the source parameter estimation becomes very low. More concretely, we calculate the entropy on the posterior probability function, which indicates the amount of uncertainty, and, when it goes below a certain threshold, the algorithm supposes to have enough information to stop and start the source declaration procedure.

Additionally, there is also a timeout that forces the algorithm to stop when the estimation takes more time than expected. In the global framework; this timeout is represented by a pre-established total number of iterations and, in the local framework, it corresponds to a given maximal number of local maps.

## 3. Evaluation

To validate the performance of the algorithm, we first implemented and evaluated the method in the global framework, both in simulation and in physical experiments using a wheeled mobile robot. The same procedure was then carried out for the evaluation of the method in the local framework.

### 3.1. Global Framework

We carried out systematic experiments in both simulation and physical reality to study the impact of some algorithmic parameters as well as environmental conditions. In the present section, we explain the materials, the experimental procedure, and the outcome.

#### 3.1.1. Simulation Experiments

**Figure 4:**
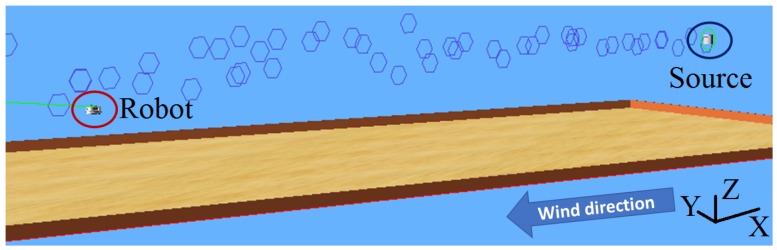
Simulation environment in Webots, with the source upwind, the robot downwind, and the odor patches represented with blue hexagons.

For the simulation experiments to be as realistic as possible, we used Webots [28], which is an open-source high-fidelity robotics simulation software. Our simulation environment was extended with an odor dispersion plugin [29] which allows for a reasonably realistic simulation of wind and odor plume, based on the filament-based atmospheric dispersion model proposed in [1]. The simulated wind flow is quasi-laminar and stationary in its intensity and direction (i.e., no meandering and wind gusts). We also used a simulated Khepera III robot, equipped with an olfaction and anemometer sensor. A view of the simulated environment can be seen in Figure 4.

In this particular setup, by disabling the gravity in the software, we were able to use our Khepera III robot as an aerial robot to assess the algorithm in 3-D. To make the performance evaluation as fair as possible, we randomly set the initial position of the robot, as well as the position of the source on *Y* and *Z* axes. On *X*, the source remains on extreme upwind direction, with 1 m distance from the wall. This choice ensures that the setup is as challenging as possible.

**Figure 5:**
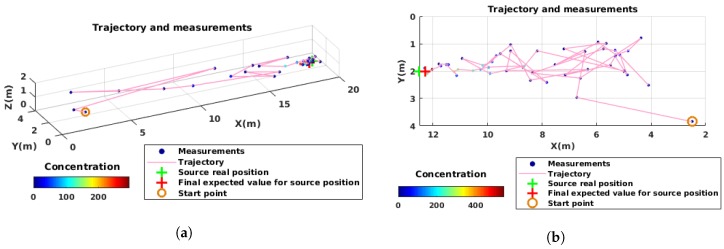
Two examples of robot’s trajectory in the global framework: (**a**) simulation; and (**b**) physical reality. The initial position of the robot, as well as the real position of the source and its predicted one are shown. On each sampled point, the measured concentration is indicated by the color of the circle, which corresponds to the averaged value of the measurements in a 5-s time window. The measured concentrations are relative values given by the sensor in mV.

Figure 5a shows a sample trajectory of the robot. In this example, the robot started far from the source. At the beginning of the experiment, large steps were taken to explore the environment. When the uncertainty gradually decreased, the maximum a posteriori value converged to the expected value and as a result the steps became smaller. Finally, for the uncertainty to become negligible, the robot sampled several points around the source position area before stopping the algorithm.

##### 3.1.1.1. Algorithmic Parameters

The presented algorithm has three essential parameters that impact the performance: model and measurement errors considered in the likelihood, $\sigma_{M}$ and $\sigma_{D}$ in Equation (4), that influence the uncertainty on the estimation and the ratio α between exploration and exploitation in the navigation part. In the simulation, we assumed to have no measurement error (i.e., $\sigma_{D}=0$), firstly because in our simulation we could set the measurement noise to 0 and secondly because we wanted to calculate the model error $\sigma_{M}$ in our setup. The value of measurement error $\sigma_{D}$ was studied in physical experiments, as presented in Section 3.1.2. In the simulation, we evaluated the performance of the algorithm using different values of the two remaining parameters to find the best set of values.

The metric that we used for evaluation is the number of iterations (or number of samples) that it takes the robot to reach the end of the algorithm as well as the estimation error on the source position on the *X*-, *Y*-, and *Z*-axis. The algorithm estimates other parameters of the model as well, namely *Q*, $\sigma_{y}$ and $\sigma_{z}$, but their values are not of primary interest for us as we focused on the performance of the robot as opposed to the faithfulness of the model to the physical transport phenomenon. Both metrics deliver positive values, with the lower the value, the better the performance. For each set of values, we repeated the simulation 10 times in a fixed environmental condition, where the wind speed was set to 0.9 m/s and the source release rate to 5%. This condition is labeled C in Table 1 and the actual values are mentioned in Table 2.

**Figure 6:**
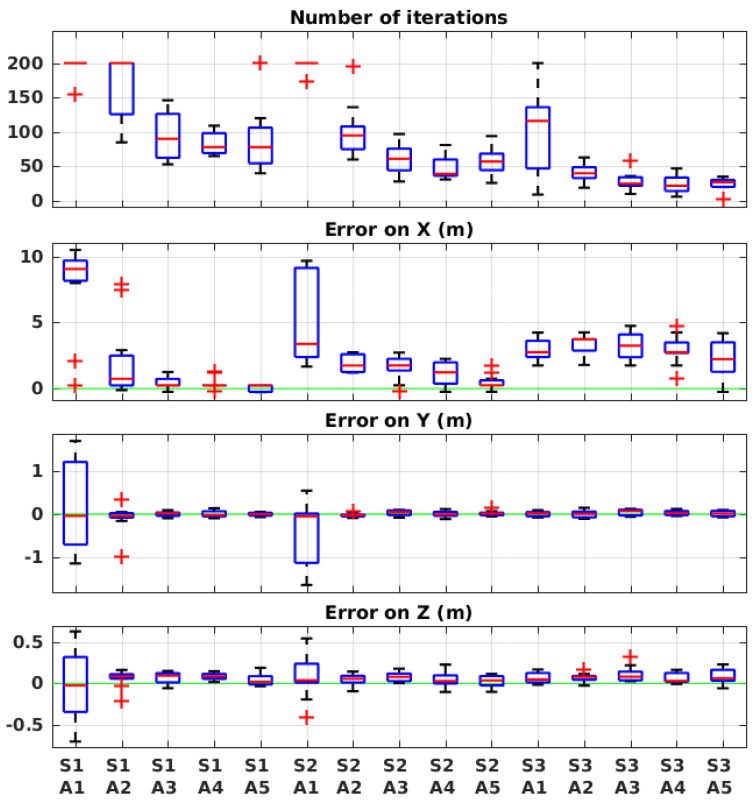
Evaluation results in simulation with different algorithmic parameters in the global framework. See Table 3 for the meaning of the labels.

The results are presented in Figure 6. On each box, the central mark indicates the median, and the bottom and top edges of the box indicate the 25th and 75th percentiles of the results, respectively. The whiskers extend to the most extreme data points without considering the outliers, which are plotted individually using the “+” symbol. The parameter values corresponding to the labels are presented in Table 3. The results show that the likelihood model error $\sigma_{M}$ has a direct impact on the number of iterations. Naturally, the more uncertainty is considered, the more time the algorithm needs to converge. However, too low values also have negative impact on the estimation of the *X* value of the source position. The justification is that the pseudo-Gaussian plume model that we use in our algorithm does not entirely match the model used in the simulation odor dispersion plugin. Therefore, we need to allow for some values of model error. Given the results, $\sigma_{M}=30$ seems to be the best compromise between the two metrics.

As for the exploration–exploitation ratio α, when at the highest value A1 (high exploration), it visibly perturbs the performance. On lower values (A2–A5), on the other hand, it appears to affect mostly the number of iterations, but very slightly. Therefore, we chose the middle value 0.5, which means 50% ratio between exploration and exploitation.

##### 3.1.1.2. Environmental Parameters

Once the best algorithmic parameters were deduced, we studied the performance of the method in different environmental conditions. We considered two environmental parameters that have a high impact on the complexity of the problem: wind speed and source release rate. Indeed, when the wind speed is high, the plume is narrow, i.e., the crosswind section of the plume is small. Therefore, once the plume is found by the robot, following it towards the source is relatively easy. When the wind speed is low, on the other hand, the plume takes a less defined shape, its crosswind section becomes larger and the robot has a higher chance of failing in its task. Additionally, for a given source release rate, when the wind speed is low, due to diffusion, the gas becomes more dispersed in the environment and thus the concentration becomes lower at a given point located at the center of the plume axis, compared to a situation where the wind speed in high. Therefore, the contrast between two points that are inside and outside of the plume becomes less significant in low wind conditions. Furthermore, since the source release rate impacts the intensity of the concentration levels that the robot senses, the robustness of the method to the amplitude of the odor concentration data was evaluated by varying it. Table 2 lists the values of the environmental parameters in our experiments. Each set of experiments was repeated 10 times.

**Figure 7:**
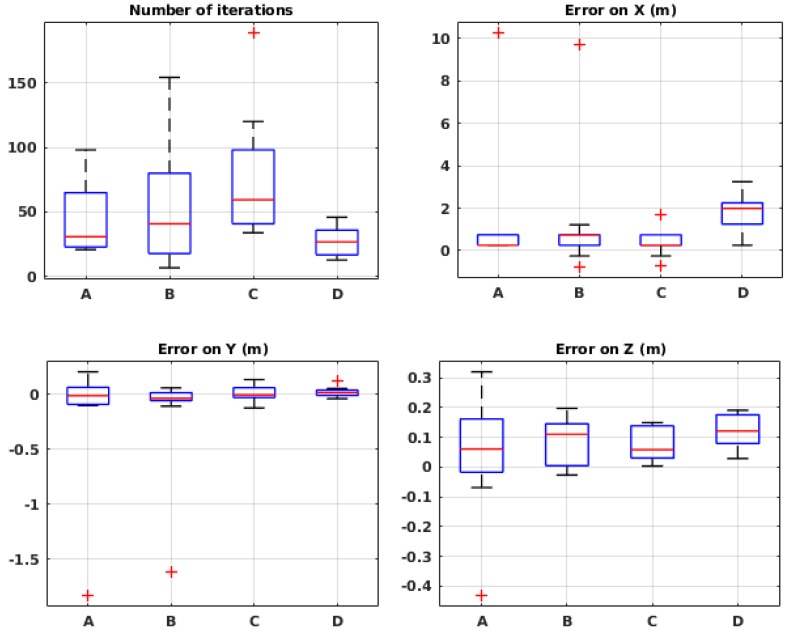
Evaluation results in simulation for different environmental conditions in the global framework. See Table 1 for the meaning of the labels.

The results of these evaluations are presented in Figure 7. Apart from a few outliers, the error is mostly very low on the source position and the number of iterations seem to be acceptable in all environmental conditions. The error is around 10 or 20 cm on *Y* and *Z* axis. On *X*-axis, on the other hand, the error is usually around 1 m. We suspect that the issue could come from the granularity for estimating the posterior probability density function, which is 0.5 m on *X*, while 0.2 m on *Y* and *Z*. From a general point of view, however, we can see that the performance of the algorithm is very similar in all the tested environmental conditions. This result was expected, since the underlying model of the experiment adapts itself to both wind speed, which is captured using an anemometer, and release rate, which is estimated during the experiment by the algorithm.

It is worth mentioning that, since a high wind speed and a high release rate make the task of OSL easier for the robot, as explained above, we expected to obtain better results in condition D (see Table 1 for the meaning of the label). This expectation was validated in the number of iterations, but not in the accuracy of the estimation, best shown by the error on the *X*-axis. This could be caused either by the fact that the algorithmic parameters were studied and adjusted only in condition C, or by the discrepancy between the plume model (pseudo-Gaussian and representing the stationary distribution of the plume) used in the algorithm and the one used in the simulation (filament based [1] and emulating the dynamics of the plume). Such discrepancy is in any case small as demonstrated in [1] and our implementation. Indeed, in [1], it is shown that the filament-based model matches the long-term time-averaged pseudo-Gaussian plume model. However, the comparison was made with a three minutes time-averaged data, and the chosen $\sigma_{y}$ and $\sigma_{z}$ were a nonlinear function in the x coordinate. In our simulation setup up, the long-term equivalence of the two models was validated with a second degree polynomial function for $\sigma_{y}$ and $\sigma_{z}$. However, for the sake of simplicity, and since the coefficient of the second degree term was negligible, we chose to approximate the equations to a first degree linear function.

#### 3.1.2. Physical Experiments

**Figure 8:**
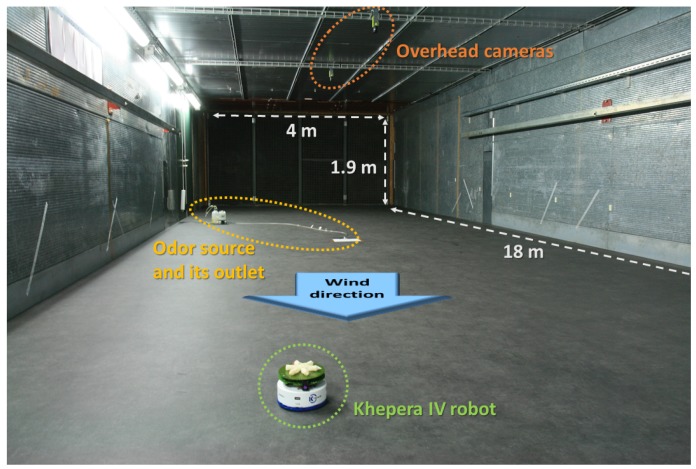
A view of the wind tunnel environment, along with a Khepera IV robot, the emulated odor source as well as the overhead cameras.

To evaluate the performance of the algorithm in a repeatable fashion, our experiments were carried out in a wind tunnel of volume $18\times 4\times 1.9m^{3}$, which provides a controllable wind flow. The wind tunnel is designed in such a way that the wind passes first through a flow straightener that consists of a set of narrow tubes of 10 cm in diameter, assembled in a honeycomb-like structure. The resulting Reynolds numbers after the honeycomb would be between 4000 to 6000 for the considered wind speed range and a temperature of $15\;{}^{\circ }$C. However, the flow gets further laminarized through a very fine-structured filter, right before entering the main chamber where experiments are carried out. Given the design of the wind tunnel and the experimentally observed data, we assumed that the resulting air flow is quasi-laminar. A Khepera IV robot, equipped with an olfaction sensor MiCS-5521 CO/VOC [30] as well as a wind sensor board [4], ran the algorithm autonomously. Overhead cameras in combination with the SwisTrack software [31] were used for tracking the robot pose in the wind tunnel, enabling both the implementation of an absolute positioning system when the localization information is sent back to the robot in the global framework algorithms and, for both algorithmic frameworks, as ground truth system for performance evaluation. The localization information was sent back to the robot to be used in the algorithm in the global framework. The odor source was emulated using an electric pump vaporizing ethanol. An illustration of the wind tunnel with the deployed equipment is presented in Figure 8. Since the mobility of the robot is only in 2-D, the source localization error was only calculated on the *X*- and *Y*-axis.

Figure 5b shows a sample trajectory of the robot in the wind tunnel. Similar to the simulation experiments, first, the robot started to explore the environment by taking large steps. Once it had found the plume and had less uncertainty about the source parameters, the steps became smaller and the overall movement was oriented towards the source. The final expected source position was only a few centimeter away from the real source position.

The concentration values show that the plume is very narrow close to the source, and therefore, a slight deviation from the plume axis results in a lower concentration. Additionally, there is a slight shift in the wind direction in the test environment, which is why the plume is not exactly aligned with *X*-axis.

##### Algorithmic Parameters

Unlike in simulation, in physical experiments, it is inevitable to have measurement errors. Therefore, in our wind tunnel experiments, we studied the parameter $\sigma_{D}$ of Equation (4), which is the standard deviation of the measurement error. In these experiments, the parameters $\sigma_{M}$ and α were fixed to the best values deduced from the simulation results presented in Section 3.1.1.1.

**Figure 9:**
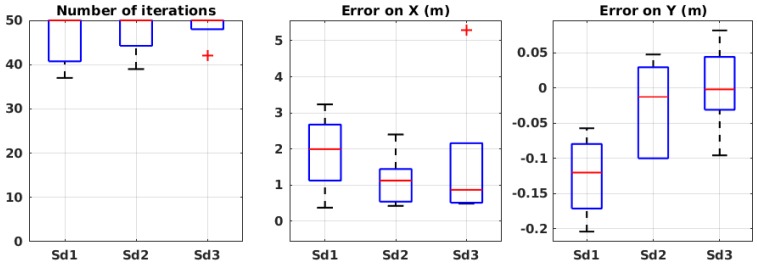
Evaluation results in wind tunnel experiments with different values of $\sigma_{D}$. See Table 3 for the meaning of the labels.

Figure 9 shows the performance of the algorithm with different values of $\sigma_{D}$. In these experiments, the maximum number of iterations was fixed to 50. With $\sigma_{D}=150$, the error on *X* and *Y* are very small, but the number of iterations is very close to 50. When $\sigma_{D}=100$ the error on *X* is about 2 m on average, which is relatively high. This is why the middle value, 125, was chosen for the rest of the experiments.

##### Environmental Parameters

Similar to the simulation, the algorithm was also evaluated in the environmental conditions listed in Table 2. Each set of experiment was carried out five times.

**Figure 10:**
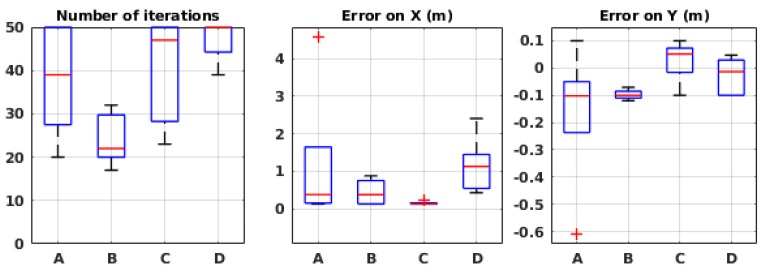
Evaluation results in wind tunnel experiments in different environmental conditions. Refer to Table 1 for the meaning of the labels.

Figure 10 shows the results of the experiments in different environmental conditions. In most of the experiments, there is a larger error on *X* compared to *Y*, similar to the simulation results. In these experiments, the amount of error is higher compared to the simulation, mainly because the maximum number of iterations was arbitrarily fixed to 50, but also because of different irregularities that happen in physical experiments. In all cases, the mean error on *X* is less than 1.2 m and on *Y* less than 0.1 m. Therefore, we can conclude that the algorithm shows robustness to the wind speed and source release rate, in the tested configurations. Further evaluations in more extreme conditions would be necessary to claim that the algorithm is robust to any environmental condition.

### 3.2. Local Framework

Once the method was validated in the global framework, we also evaluated it in the local one, again first in simulation and then in wind tunnel, in the very same setups used for the global framework algorithm. The underlying algorithm and experimental environments being identical in both frameworks, we used the chosen values of the parameters that we previously studied, namely the exploration–exploitation ratio α and the likelihood’s model and measurement errors $\sigma_{M}$ and $\sigma_{D}$.

#### 3.2.1. Simulation Experiments

For the local framework evaluation, we used the same simulation environment in Webots as in the global framework described in Section 3.1.1, only in 2-D. Due to the characteristics of the framework, the Khepera III robot localized itself using odometry, and its navigation and estimation field was limited to the local map, shown by the green square in Figure 2. The size of the local map $2\times 2\;m^{2}$ was chosen in order to cover a small portion of our $20\times 4\;m^{2}$ arena, and, at the same time, to leave enough space for the robots to explore.

**Figure 11:**
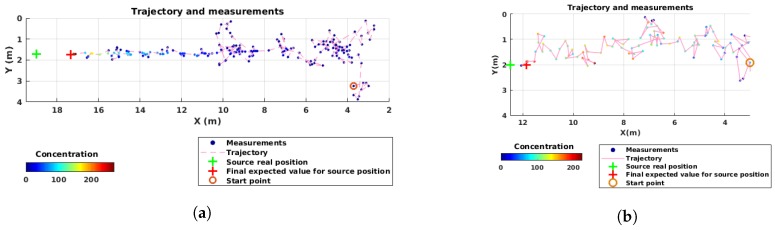
Two examples of the robot’s trajectory in the local framework: (**a**) simulation; and (**b**) wind tunnel. The initial position of the robot is shown with an orange circle; the actual source position (green cross) as well as its estimated location (red cross) are also indicated. On each sampled point, the measured concentration is indicated by the color of the circle, which corresponds to the averaged value of the measurements in a 5-s time window. The measured concentrations are relative values given by the sensor in mV.

Figure 11a shows a sample trajectory of the robot in simulation. The samples are shown along with the robot’s trajectory. As expected, the robot took small steps and changed its course usually towards the source position. At the end, the robot stopped very close to the source where the algorithm reached its end.

##### Algorithmic Parameters

As mentioned above, three algorithmic parameters were studied in the global framework, and we simply used their chosen values for the local framework. However, in the local framework, we introduced a new algorithmic parameter, namely the initial sample budget value $B_{0}$, that we still needed to study.

**Figure 12:**
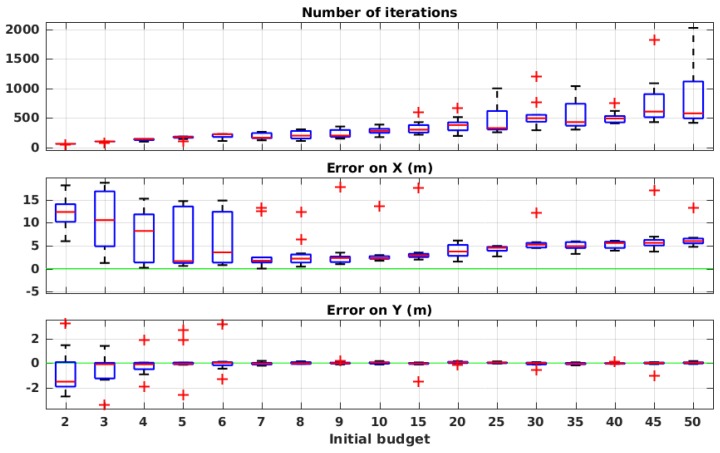
Evaluation results in simulation with different initial values of the sample budget.

Figure 12 shows the performance of the method with different values of $B_{0}$, each repeated 10 times. As expected, the number of iterations systematically increases with higher values of the initial budget. The source position estimation error, on the other hand, shows different results: with very low values of sample budget, the error is very high, since the algorithm does not obtain enough information to guide the robot in an efficient way. With values between 7 and 9, the error drops to acceptable values (1 to 2 m of error on *X*). For larger values, however, such as 25–50, we notice a slight increase in the error value on *X*. We believe that this error is due to accumulated odometry error after so many steps. For the rest of the evaluation, we set the initial budget value to 7, which seems to be a close to optimal value, according to the presented results.

##### Environmental Parameters

To assess the robustness of the method to environmental conditions, we evaluated the performance of our method in the local framework in the same conditions as in the global framework, which are listed in Table 2.

**Figure 13:**
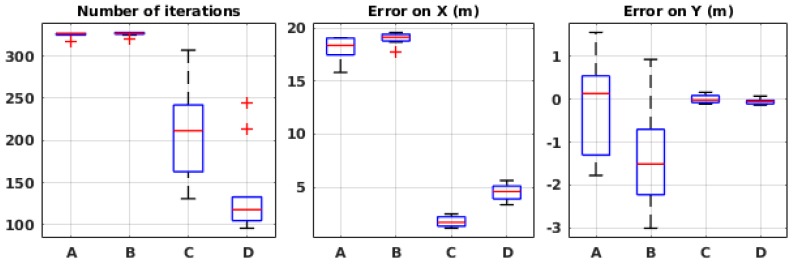
Evaluation results in simulation for different environmental conditions in the local framework. See Table 1 for the meaning of the labels.

Figure 13 presents the performance of the system in 10 runs for each environmental condition. While showing satisfactory results in some situations, the algorithm fails in the others. It appears that, in high wind speed (conditions C and D), the error is in an acceptable range, but, in low wind speeds, the algorithm fails. We believe that it is due to the limited estimation field. Indeed, when the robot is far from the source, in some situations, the posterior probability distribution created on the local map predicts in which orientation the source is located and therefore guides the robot towards it. In low wind speed, on the other hand, this does not happen because the plume is much wider and thus it guides the robot less accurately, and, since the robot does not keep the outcome of the previous local maps, it cannot recover after being misguided.

#### 3.2.2. Physical Experiments

**Figure 14:**
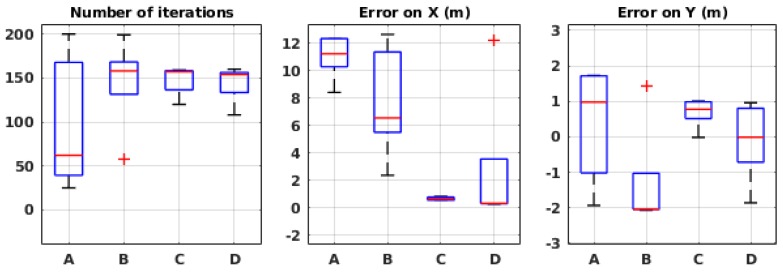
Evaluation results in wind tunnel experiments for different environmental conditions in the local framework. See Table 1 for the meaning of the labels.

The algorithm was also tested in the local framework in a wind tunnel. The same setup and environmental conditions were used as in the global framework. The results, as shown in Figure 14, seem consistent with the ones from the simulation in Figure 13. Indeed, in high wind speed, the mean error is 0.25 m on the *X*-axis and less than 1 m on the *Y*-axis. However, again, in low wind speeds, the algorithm seems to be misguided and fails. With the confirmation of physical experiments, we know that the algorithm cannot be robust to different environmental conditions in its current implementation.

In Figure 11a, a sample trajectory of the robot in a physical experiment is shown. Similar to the sample trajectory from a simulation experiment shown in Figure 11b, the robot takes short steps and finishes the task very close to the source.

## 4. Discussion

The algorithm shows robustness to the tested environmental conditions in the global framework, but, in the local framework, it only works in high wind speed. We believe that it is because, in the global framework, the source is certainly located in the map, where the estimation takes place. In the local framework, the local map does not initially enclose the source, but, using the estimated posterior distribution, it supposedly guides the robot towards the source.

**Figure 15:**
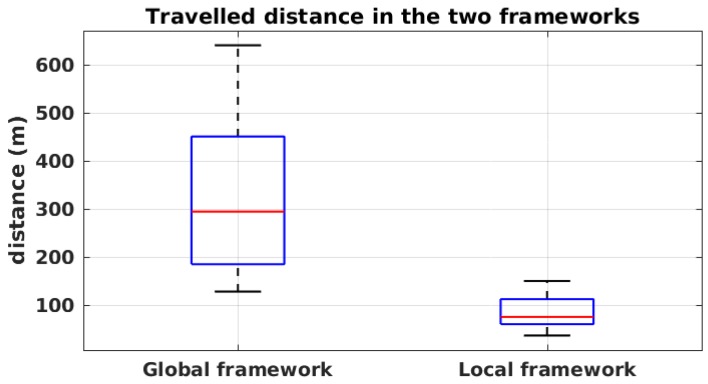
Travelled distance in simulation for the environmental condition C in both frameworks.

One advantage of the local framework that we did not address is the low travelled distance. In Figure 15, the total travelled distances in both frameworks are shown in simulation in condition C. It appears that, in the local framework, on average, the robot moves three times less than in the global framework. The limited navigation strategy in the local framework, on the one hand, and the large exploration steps in the global framework, on the other hand, are the causes of the difference between the two frameworks.

In one of our previous works [32], proposing a 3-D odor source localization algorithm based on Infotaxis, a similar structure to the present work was used. They both rely on an odor plume model, but, in the Infotaxis algorithm, the model with all its parameters is assumed to be known before the experiment. This assumption, while being applicable for a known environment, limits the usage of the method in real world scenarios where there is usually no a priori information about the plume. The STE-based algorithms, on the other hand, do not use a priori information on the parameters of the model and estimate the values based on the acquired data. The only predefined values are the limits on the range of each parameter values. Thus, by choosing realistic yet meaningful ranges, the algorithm could be run in different configurations.

It is worth mentioning that a few assumptions are included in this STE algorithm, as explained in Section 2.3. However, they are different from a priori information which are meant to simplify the evaluation. In this work, by making assumptions, we did not seek to simplify the evaluation of the algorithm, but only the algorithm itself and its computation. For example, assuming that the plume is isotropic (i.e., $\sigma_{y}=\sigma_{z}$) was meant to decrease the number of parameters to estimate, hence to simplify the computation. Such simplifications might not match with the real setup and thus the algorithm would work less efficiently. However, since the algorithm showed success with these limitations, we can expect that it would work more efficiently without them. The same reasoning is valid for the choice of the model, and the assumption based on which it is driven.

Another interesting difference between this work and our previously mentioned one [32] is that, in that work, the estimation was done on the entire map of the environment (globally) while the navigation was done in small steps, i.e., more similar to our local framework. The results of that work showed lower performance on low wind speed as well compared to high wind speeds. However, the mean of error on source localization was lower in [32] than in the present paper. Therefore, a combination of local navigation and global estimation seems to be more suitable for this type of algorithms.

## 5. Conclusions

We successfully developed an odor source localization algorithm based on the STE method. The algorithm was evaluated in two frameworks, global and local, in both simulation and physical reality. The impact of different algorithmic parameters were studied and the robustness of the method to different environmental configurations, namely wind speed and source release rate, was evaluated.

One of the advantages of this algorithm was its ability to cover the three sub-tasks of odor source localization problem. The enhanced navigation methods proposed in this paper allowed for an efficient search for the plume acquisition part. The amount of uncertainty associated with the estimation ensured the source validation sub-task as well.

Thanks to the MCMC method, the high computational cost related to probabilistic algorithms was remarkably reduced and the algorithm could be run entirely on a Khepera IV robot.

The successful evaluation of this method in the local framework, without any predefined environment map and any global positioning system, shows the high potential of this algorithm for unknown environments. However, its downside is the lack of robustness to different environmental conditions, which could be studied in future work.

## Figures and Tables

**Table 1:** Environmental condition setups.

Label	A	B	C	D
Wind Speed	Low	Low	High	High
Release Rate	Low	High	Low	High

**Table 2:** Environmental parameters studied in simulations and wind tunnel experiments.

Parameter	Tested Values
Wind speed (m/s)	Simulation: 0.2 (low), 0.9 (high)
	Wind tunnel: 0.6 (low), 1.0 (high)
Source release rate	Simulations: 5% (low), 10% (high)
	Wind tunnel: 70% (low) and 100% (high)

**Table 3:** Labels associated with algorithmic parameters value.

Label	S1	S2	S3		Label	Sd1	Sd2	Sd3		Label	A1	A2	A3	A4	A5
$\sigma_{M}$	50	30	10		$\sigma_{D}$	100	125	150		α	0.9	0.7	0.5	0.3	0.1

## Abbreviations

The following abbreviations are used in this manuscript:

OSL	Odor Source Localization
STE	Source Term Estimation
